# Bile salt hydrolase of *Lactiplantibacillus plantarum* plays important roles in amelioration of DSS-induced colitis

**DOI:** 10.1016/j.isci.2023.106196

**Published:** 2023-02-13

**Authors:** Xin Feng, Zichen Bu, Hongyu Tang, Yongjun Xia, Xin Song, Lianzhong Ai, Guangqiang Wang

**Affiliations:** 1 Shanghai Engineering Research Center of Food Microbiology, School of Health Science and Engineering, University of Shanghai for Science and Technology, Shanghai 200093, China

**Keywords:** Microbiology, Microbiome, Cell biology

## Abstract

Bile salt hydrolases are thought to be the gatekeepers of bile acid metabolism. To study the role of BSH in colitis, we investigated the ameliorative effects of different BSH-knockout strains of *Lactiplantibacillus plantarum* AR113. The results showed that *L. plantarum* Δ*bsh* 1 and Δ*bsh* 3 treatments did not improve body weight and alleviate the hyperactivated myeloperoxidase activity to the DSS group. However, the findings for *L. plantarum* AR113, *L. plantarum* Δ*bsh* 2 and Δ*bsh* 4 treatments were completely opposite. The double and triple *bsh* knockout strains further confirmed that BSH 1 and BSH 3 are critical for the ameliorative effects of *L. plantarum* AR113. In addition, *L. plantarum* Δ*bsh* 1 and Δ*bsh* 3 did not significantly inhibit the increase in pro-inflammatory cytokines or the decrease in an anti-inflammatory cytokine. These results suggest that BSH 1 and BSH 3 in *L. plantarum* play important roles in alleviating enteritis symptoms.

## Highlights

• AR113 could alleviate the disease of mice with DSS-induced colitis
• *L. plantarum* Δ*bsh* 1 and Δ*bsh* 3 could not regulate inflammatory cytokine-related genes
• *Bsh* 1 and *bsh* 3 are critical for ameliorative effects in colitis

## Graphical abstract

**Figure:**
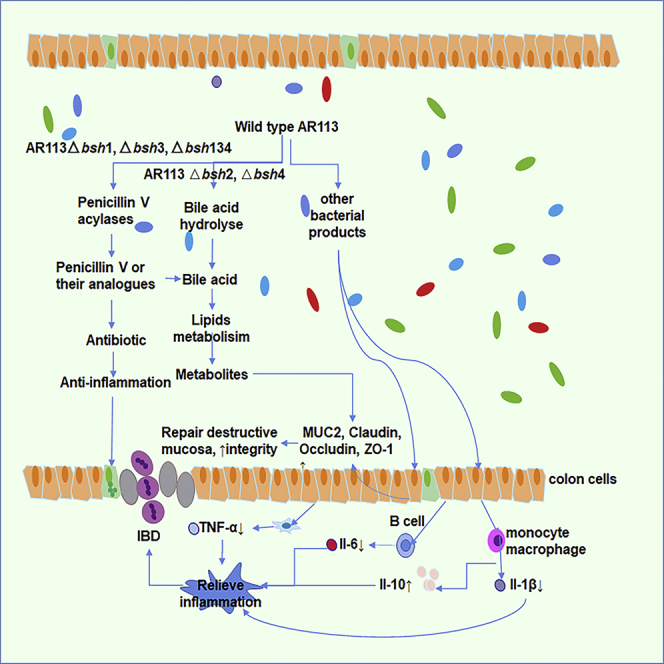

## Introduction

Inflammatory bowel diseases (IBDs) are mainly divided into ulcerative colitis (UC) and Crohn’s disease, the typical symptoms of which include abdominal pain, diarrhea, blood in the stool, loss of appetite, fatigue, and weight loss.^1^ The incidence and prevalence of IBD have increased markedly in recent years, making IBD a global public health problem, which has attracted worldwide attention. Although the exact cause of IBD remains largely unknown, several studies have suggested that it involves a complex interaction between genetic, microbial, and immune factors.^1^^,^^2^^,^^3^^,^^4^^,^^5^ Recent research has revealed that of these factors, the composition of the intestinal microbiota is especially important in IBD pathogenesis.^3^^,^^4^^,^^5^

Some recent studies have suggested that the intestinal microbiota affects IBD pathogenesis through metabolites such as short-chain fatty acids (SCFAs) and bile acids (BAs).^6^^,^^7^^,^^8^^,^^9^^,^^10^^,^^11^^,^^12^ SCFAs metabolized by intestinal bacteria have been shown to ameliorate the typical symptoms in animal models of IBD and have been associated with a reduced risk of IBD.^7^^,^^8^^,^^9^^,^^10^ In addition, some recent studies have found that changes in BAs and BA metabolism are also linked to IBD.^10^^,^^11^^,^^12^^,^^13^ Studies have found that although the levels of primary BAs and conjugated BAs (CBAs) are augmented in the fecal samples of healthy individuals, the levels of secondary BAs are decreased in the fecal samples of IBD patients, however, it is not clear why IBD patients manifest these changes and what role these changes play in IBD pathogenesis.^11^^,^^12^ Intestinal microorganisms can promote the conversion of primary BAs to secondary BAs, thus changing the composition of BAs in the body. As bile salt hydrolases (BSHs) can cleave the amide bond in conjugated BAs to increase the concentration of deconjugated BAs, which can subsequently undergo a variety of transformations to generate secondary BAs, they are thought to be the gatekeepers of BA metabolism and host microbiome crosstalk in the gastrointestinal tract.^14^^,^^15^ Labbe et al.^13^ found a significant reduction in the abundance of the Firmicutes-derived BSH (*bsh*) gene in IBD patients relative to healthy controls, using large available datasets containing metagenomic information from IBD patients. Studies have also reported a significant negative correlation between the relative abundance of bacterial *bsh* genes and the retinoic acid receptor-related orphan receptor gamma (RORC) gene, which is related to IBD.^11^^,^^16^ Using chemoproteomic approaches, Parasar et al.^17^ identified altered BSH activities in a murine model of IBD. In silico analysis showed that the relative abundance of BSH in the gut microbiota was markedly lower in IBD patients than in healthy individuals, and that this reduction was most evident in Firmicutes from the patients.^17^ Although an association of BSH in the gut microbiota with IBD has been suggested, further research is warranted to provide the evidence.

Current treatment strategies for IBD focus on reducing the inflammatory burden in patients with active disease and maintaining remission in those with inactive disease.^18^^,^^19^ However, they are associated with a wide range of possible severe side effects, and some of these treatments are costly.^18^^,^^19^^,^^20^ As a result, probiotics are attracting increasing research interest as a safer way of ameliorating disease activity.^20^ Several studies have found that some probiotics can ameliorate or prevent IBD.^20^^,^^21^^,^^22^^,^^23^^,^^24^ Although the mechanisms of the ameliorative effects of probiotics on IBD are not well defined, many mechanisms have been proposed to explain this action, including antagonism to pathogenic bacteria, modulation of gut microbiota, production of nutrients, and enhancement of anti-inflammatory cytokine levels.^20^^,^^21^^,^^22^^,^^23^^,^^24^ A meta-analysis showed that probiotic VSL#3 is effective in inducing remission in active UC and is equivalent to 5-aminosalicylic acid (5-ASA) in preventing UC relapse.^20^ Wang et al.^21^ found that *Lactiplantibacillus plantarum* ZS2058 was an efficient producer of conjugated linoleic acid (CLA) *in vitro* and that it ameliorated dextran sulfate sodium (DSS)-induced acute colitis by producing CLA locally in mice. *Clostridium butyricum* producing high levels of SCFAs was revealed to alleviate epithelial damage in rats with DSS-induced colitis.^22^ Ke et al.^23^ demonstrated that fucose ameliorated intestinal inflammation by regulating the crosstalk between BAs and the gut microbiota in DSS-treated mice. However, few studies have evaluated the role of BSH in the ameliorative effect of probiotics on IBD. The family *Lactobacillaceae* is rich in BSHs. A total of 551 BSHs from 107 Lactobacillaceae species were identified from 451 genomes of 158 *Lactobacillaceae* species.^24^ Through metagenomic analyses, Jones et al.^15^ demonstrated that BSH activity is a conserved microbial adaptation to the human gut environment with a high level of redundancy in this ecosystem. Therefore, it is necessary to investigate whether probiotics exert ameliorative effects on IBD through BSHs. Our previous work found that *L. plantarum* AR113 was more effective than other probiotic strains in alleviating epithelial damage, improving colon length, and maintaining the epithelial barrier integrity after DSS treatment.^25^ We also found that *L. plantarum* AR113 had the highest BSH activity among the 10 lactic acid bacterial strains tested with the plate assay. Furthermore, using in silico molecular docking, heterologous expression, and knockout experiments, we verified that the *bsh* 1 and *bsh* 3 genes were responsible for most of the BSH activity in *L. plantarum* AR113.^26^^,^^27^ To explore the roles of BSHs in IBD, here we investigated the ameliorative effects of BSH-knockout strains of *L. plantarum* AR113 on the disease severity of mice with DSS-induced colitis.

## Results

### BSH improves colitis symptoms

**Figure 1:**
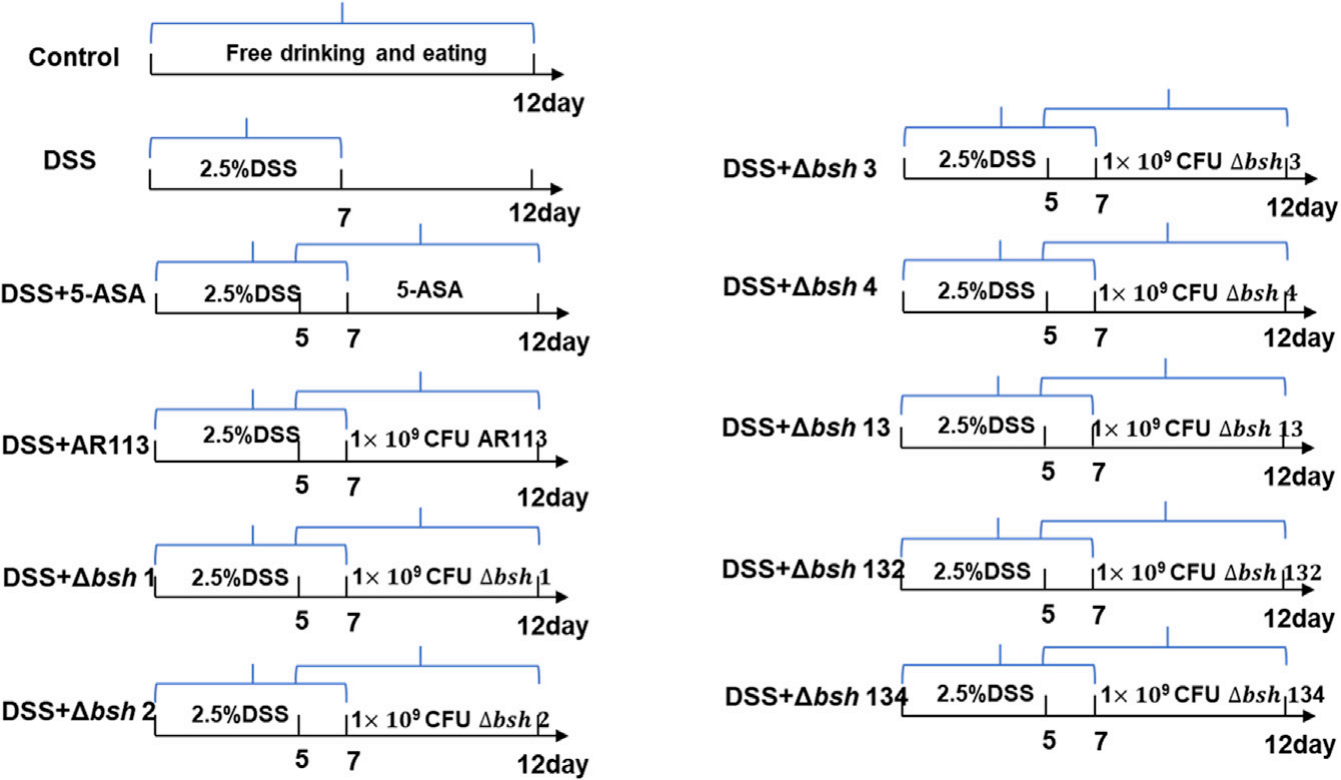
Schematic diagram of the animal experiment

To explore the roles of BSHs in IBD, the effects of orally administrated BSH-knockout strains on the severity of DSS-induced colitis in mice were evaluated based on body weight loss, DAI, colon length, MPO activity, and histopathology. Mice were fed according to the protocol outlined in Figure 1.

**Figure 2:**
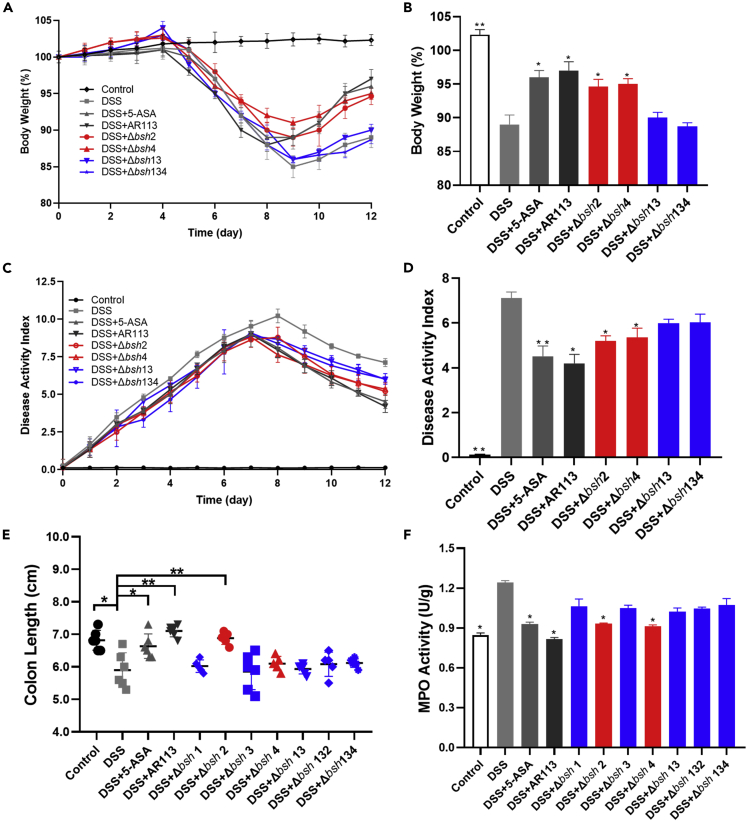
Physical and chemical indicators of colitis in mice (A) Body weight, (B) body weight at day 12, (C) disease activity index (DAI), (D) DAI at day 12, (E) colon length, (F) myeloperoxidase (MPO) activity. The red bar indicates the group with *bsh* 2 or *bsh* 4 knocked out, and the blue bar indicates the group with *bsh* 1 or *bsh* 3 knocked out. The statistical significance between the data was assessed using One-way ANOVA by Dunnett’s tests, ^∗^: p< 0.05, ^∗∗^: p< 0.01. All data are presented as the mean ± standard error of the mean (n = 8 mice per group).

The DSS group showed severe body weight loss from day 4 and demonstrated a final weight loss of 15% relative to the Control group. In the *L. plantarum* AR113 group, the DSS-treated mice showed evident recovery of body weight, with their weights being almost the same as those in the 5-ASA group and even approaching the Control group (Figure 2A). Compared with *L. plantarum* AR113, *L. plantarum* Δ*bsh* 1 and *L. plantarum* Δ*bsh* 3 led to little recovery of body weights. However, similar to *L. plantarum* AR113, *L. plantarum* Δ*bsh* 2 and *L. plantarum* Δ*bsh* 4 effectively ameliorated the body weight loss of DSS-treated mice (Figures 2A and S1A). Further analysis revealed that the *L. plantarum* Δ*bsh* 13, *L. plantarum* Δ*bsh* 132, and *L. plantarum* Δ*bsh* 134 groups had significant body weight loss with no recovery, similar to that in the DSS group (Figures 2B and S1B). These findings suggest that BSH 1 and BSH 3, not BSH 2 and BSH 4, in *L. plantarum* AR113 play important roles in the recovery of body weight in DSS-treated mice.

Compared with the DSS group, the *L. plantarum* AR113 group showed significantly reduced DAI values (10.21 ± 0.15 vs 4.20 ± 0.40, p< 0.05) and the *L. plantarum* Δ*bsh* 2 and *L. plantarum* Δ*bs*h 4 groups also showed alleviated symptoms and significantly reduced DAI values (Figures 2C, 2D and S1C). However, the DAI values of the *L. plantarum* Δ*bsh* 1 (6.28 ± 0.26), *L. plantarum* Δ*bsh* 3 (5.80 ± 0.23), *L. plantarum*Δ*bsh* 13 (6.00 ± 0.16), *L. plantarum* Δ*bsh* 13 (5.81 ± 0.22), and *L. plantarum* Δ*bsh* 134 (6.03 ± 0.17) groups did not differ significantly from one another and were significantly higher than that of the *L. plantarum* AR113 group (4.20 ± 0.40, p< 0.05, Figures 2D and S1D).

The colon lengths of mice in different groups were measured after euthanizing the mice (Figure 2E). Compared with the Control group (6.82 ± 0.31), the DSS group (5.90 ± 0.53) showed significantly decreased colon length. The *L. plantarum* AR113 treatment group (7.1 ± 0.18) effectively prevented the DSS-induced shortening of the colon and recovered the colon length to that observed in the Control and 5-ASA groups. The colon lengths in the *L. plantarum* Δ*bsh* 1, *L. plantarum* Δ*bsh* 3, *L. plantarum* Δ*bsh* 13, *L. plantarum* Δ*bsh* 132, and *L. plantarum* Δ*bsh* 134 groups were significantly reduced relative to the *L. plantarum* AR113 treatment group and were not significantly different from one another.

Figure 2F shows that the MPO activity in the DSS group (1.24 ± 0.04 U/g) was significantly higher than that in the Control group (0.85 ± 0.04 U/g), indicating that the colons of DSS-treated mice had significant neutrophil infiltration and severe inflammation. *L. plantarum* AR113 (0.82 ± 0.03 U/g) treatment reduced the DSS-induced MPO activity to almost the level in the Control group. Similar to *L. plantarum* AR113, *L. plantarum* Δ*bsh* 2 and *L. plantarum* Δ*bsh* 4 inhibited the MPO activity in DSS-treated mice. In contrast, *L. plantarum* Δ*bsh* 1 and *L. plantarum* Δ*bsh* 3 had no inhibitory effect on the MPO activity. The *L. plantarum* Δ*bsh* 13, *L. plantarum* Δ*bsh* 132, and *L. plantarum* Δ*bsh* 134 groups also showed no suppression of MPO activity relative to the DSS group. These findings suggest that *L. plantarum* AR113 uses BSH 1 or BSH 3 to alleviate the hyperactivated MPO activity in DSS-treated mice.

**Figure 3:**
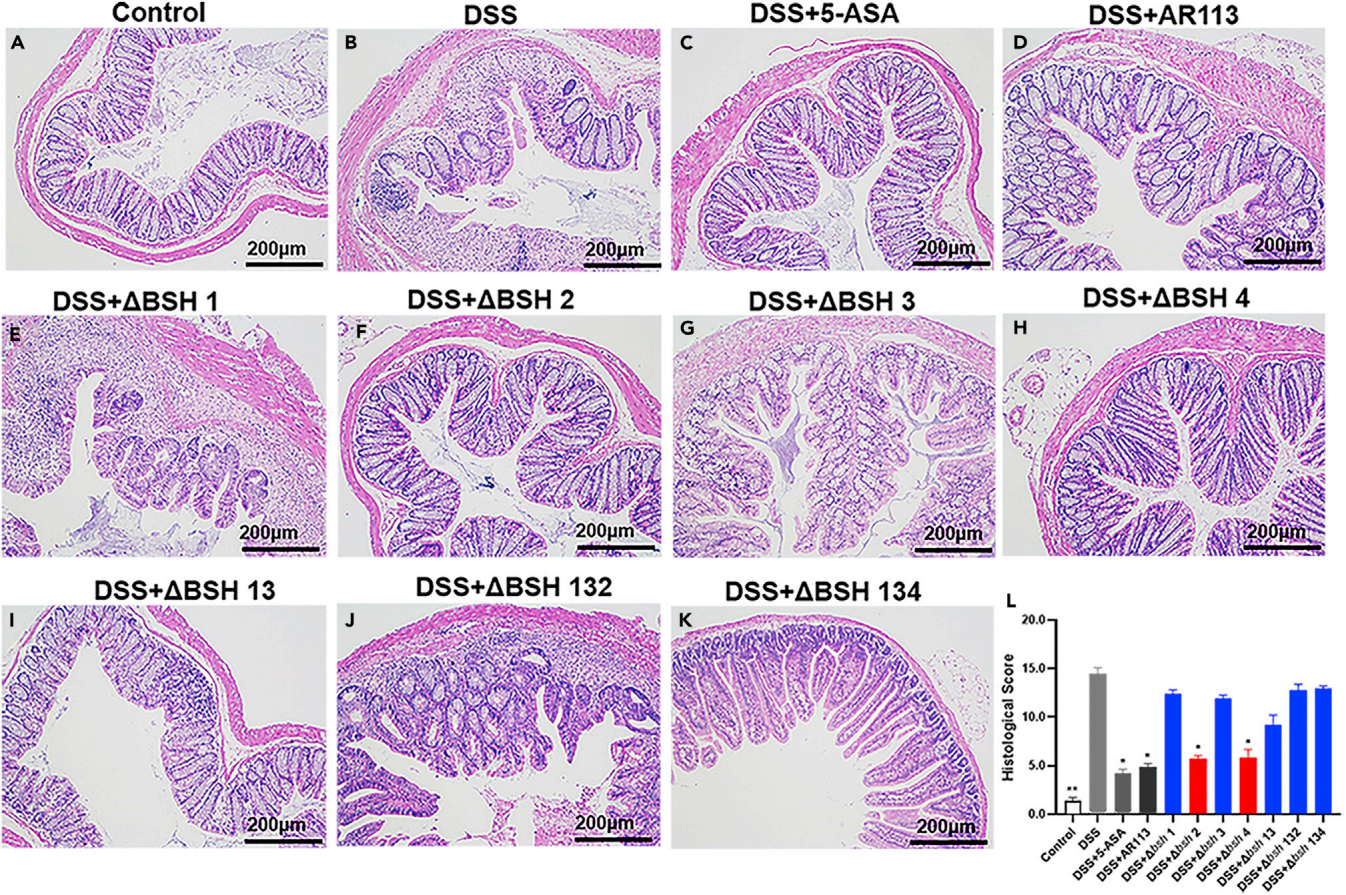
Effects of *Lactiplantibacillus plantarum* AR113 and its seven mutant strains on the colon histopathology of mice with dextran sulfate sodium (DSS)-induced colitis (A–K) Representative histological examination micrographs of colon tissue section slides. Scale bars, 200 mm. (L) Histological injury scores for the mouse colon tissues. The red bar indicates the group with *bsh* 2 or *bsh* 4 knocked out, which does not affect the function of AR113, and the blue bar indicates the group with *bsh* 1 or *bsh* 3 knocked out. The statistical significance between the data was assessed using One-way ANOVA by Dunnett’s tests, ∗: p< 0.05, ∗∗: p< 0.01.

The effect of each treatment on the pathological changes in mouse colon tissues was observed by hematoxylin and eosin (H&E) staining (Figures 3A–3K). In the Control group (Figure 3A), the intestinal epithelium was structurally intact; the complete crypt structure and many goblet cells were retained. In contrast, the DSS group showed complete destruction of the intestinal epithelial structure, loss of the crypt structure, and infiltration of a large number of inflammatory cells in the colon (Figure 3B). Similar to that in the 5-ASA group (Figure 3C), the *L. plantarum* AR113 (Figure 3D), *L. plantarum* Δ*bsh* 2 (Figure 3F), and *L. plantarum* Δ*bsh* 4 (Figure 3H) groups showed significant alleviation of the DSS-induced colon tissue lesions, with histological injury scores of 4.86 ± 0.34, 5.67 ± 0.87, 5.34 ± 0.38, and 4.23 ± 0.40 (all p< 0.05), respectively, which were significantly lower than the score of the DSS group (Figure 3L). In contrast, the *L. plantarum* Δ*bsh* 1, *L. plantarum* Δ*bsh* 3, *L. plantarum* Δ*bsh* 13, *L. plantarum* Δ*bsh* 132, and *L. plantarum* Δ*bsh* 134 groups showed destruction of the intestinal epithelial structure, loss of the crypt structure, and colonic infiltration of inflammatory cells, similar to those in the DSS group.

### Effect of BSHs on the inflammatory cytokine expression

**Figure 4:**
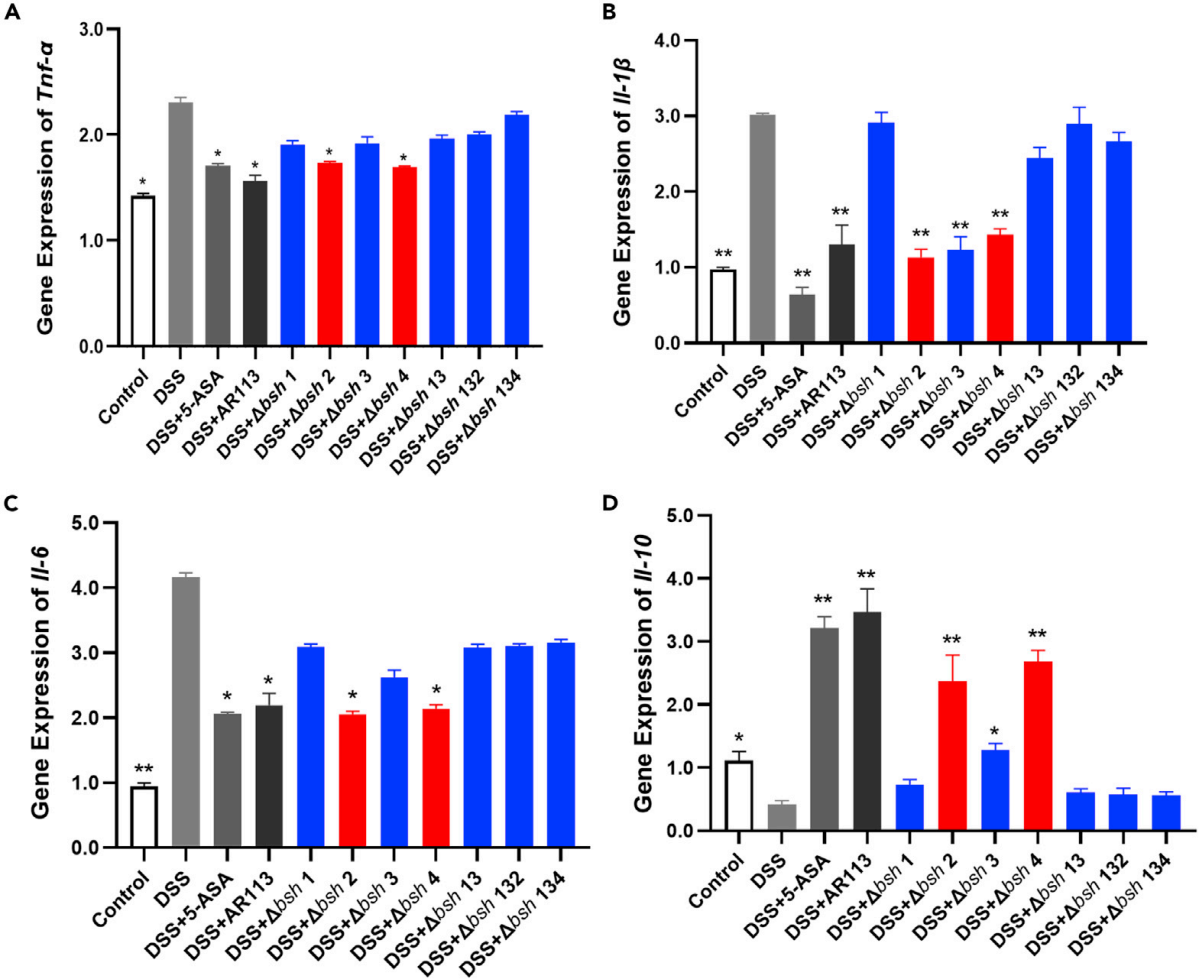
mRNA expression of the cytokines (A) Tumor necrosis factor alpha (TNF-α), (B) interleukin (IL)-1β, (C) IL-6, and (D) IL-10 was quantified by RT-qPCR. The data are expressed as the mean ± standard deviation (n = 8 mice per group). The red bar indicates the group with *bsh* 2 or *bsh* 4 knocked out, which does not affect the function of AR113, and the blue bar indicates the group with *bsh* 1 or *bsh* 3 knocked out. The statistical significance between the data was assessed using One-way ANOVA by Dunnett’s tests, ∗: p< 0.05, ∗∗: p< 0.01.

Changes in the levels of pro-inflammatory cytokines (TNF-α, IL-1β, and IL-6) and the anti-inflammatory cytokine IL-10 in mouse colon tissues were measured to determine the anti-inflammatory effect of *bsh*. The sequences of primers are listed in Table 1. As shown in Figure 4, compared with the Control group, the DSS group demonstrated a significant increase in the transcript levels of the colonic pro-inflammatory cytokines TNF-α, IL-1β, and IL-6, but a significant decrease in the transcript level of the anti-inflammatory cytokine IL-10. Similar to the 5-ASA group, the *L. plantarum* AR113 group showed significantly downregulated expression of the pro-inflammatory cytokines and upregulated expression of the anti-inflammatory cytokine relative to the DSS group. Similar to *L. plantarum* AR113, *L. plantarum* Δ*bsh* 2 and *L. plantarum* Δ*bsh* 4 also downregulated the expression of the pro-inflammatory cytokines and upregulated the expression of the anti-inflammatory cytokine. This indicates that BSH 2 and BSH 4 do not affect the inflammatory cytokine expression. In contrast to the *L. plantarum* AR113 group, the *L. plantarum* Δ*bsh* 1 and *L. plantarum* Δ*bsh* 3 groups showed no significant difference in the expression of most of inflammatory cytokines relative to the DSS group. Furthermore, the cytokine expressions in the *L. plantarum* Δ*bsh* 13, *L. plantarum* Δ*bsh* 132, and *L. plantarum* Δ*bsh* 134 groups were not significantly different from one another or from those in the DSS group. These findings suggest that BSH 1 and BSH 3 in *L. plantarum* AR113 play crucial roles in regulating the inflammatory cytokine expression in the colon tissues of DSS-treated mice.

**Table 1:** Primers sequences use in this study

Primers	Forward primers (5′-3′)	Reverse primers (5′-3′)
RFPinsert	TAGGACTAACTCTACCGAAG	CAGTCTTGTTCGGATTAATC
β-actin	GGCTGTATTCCCCTCCATCG	CCAGTTGGTAACAATGCCATGT
TNF-α	AGGGTCTGGGCCATAGAACT	CCACCACGCTCTTCTGTCTAC
IL-6	GAGGATACCACTCCCAACAGACC	AAGTGCATCATCGTTGTTCATACA
IL-1β	CTGAACTCAACTGTGAAATGC	TGATGTGCTGCTGCGAGA
MUC2	ATGCCCACCTCCTCAAAGAC	GTAGTTTCCGTTGGAACAGTGAA
Claudin1	CTGTGGATGTCCTGCGTTTC	TCATGCACTTCATGCCAATG
Occludin	TGGCGGATATACAGACCCAA	CGATCGTGGCAATAAACACC
ZO-1	CTTCTCTTGCTGGCCCTAAAC	TGGCTTCACTTGAGGTTTCTG
FGFR4	GTGGTCAGTGGGAAGTCTGG	TTGTACCAGTGACGACCACG
FGF15	GACTGCGAGGAGGACCAAAA	CAGCCCGTATATCTTGCCGT
CYP7A1	AACAACCTGCCAGTACTAGATAGC	GTGTAGAGTGAAGTCCTCCTTAGC
SHP	TCTGCAGGTCGTCCGACTATTC	AGGCAGTGGCTGTGAGATGC
FXR	TGGGCTCCGAATCCTCTTAGA	TGGTCCTCAAATAAGATCCTTGG

### Effect of BSH on the expression of tight junction (TJ)-related genes

**Figure 5:**
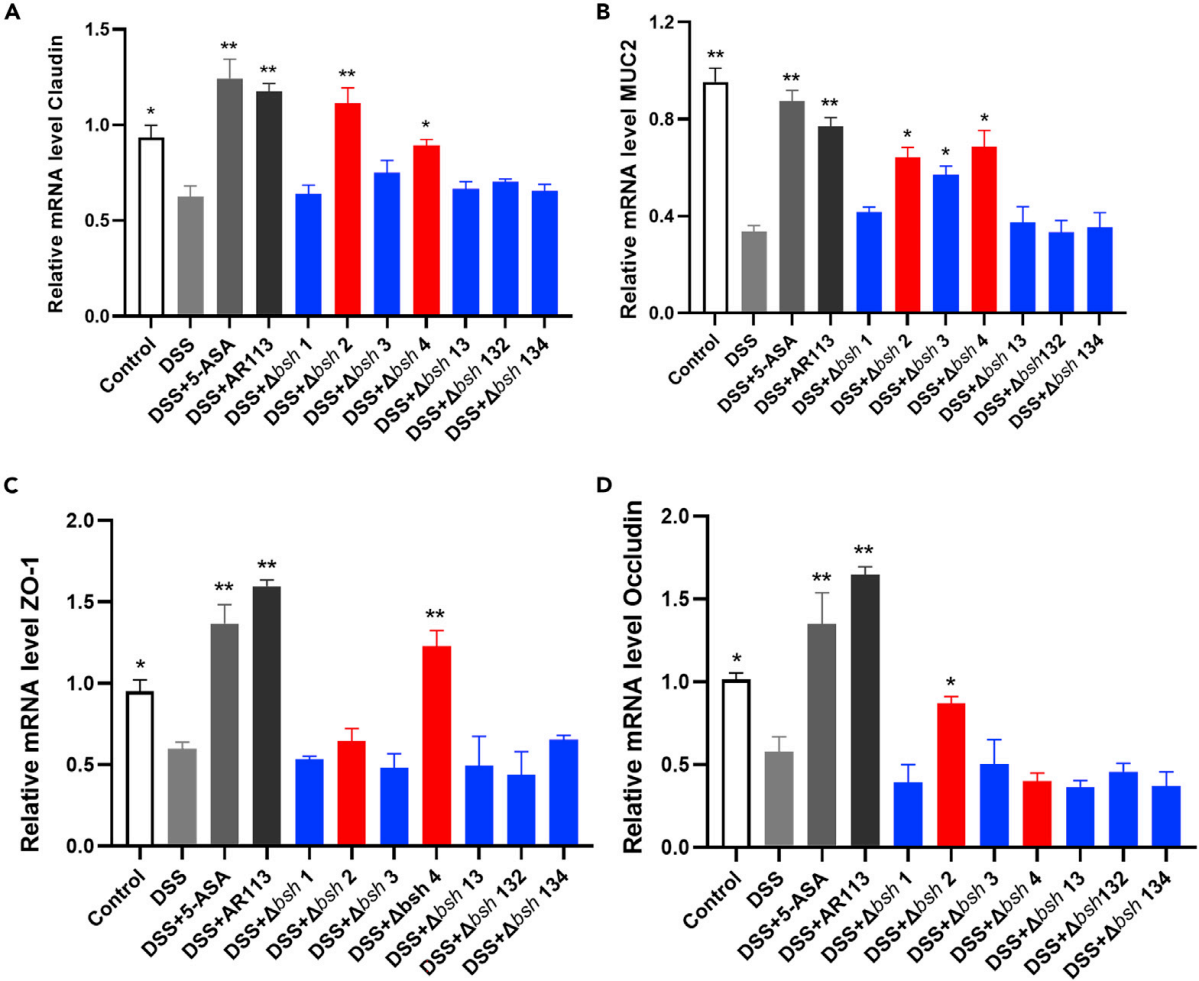
Expression of intestinal barrier function-related genes in mice (A) Claudin, (B) MUC2, (C) occludin, (D) ZO-1. The statistical significance between the data was assessed using One-way ANOVA by Dunnett’s tests, ^∗^: p< 0.05, ^∗∗^: p< 0.01. All data are presented as the mean ± standard error of the mean (n = 8 mice per group). The red bar indicates the group with *bsh* 2 or *bsh* 4 knocked out, and the blue bar indicates the group with *bsh* 1 or *bsh* 3 knocked out.

The relative expression of MUC2 secreted by the mouse colon tissues was analyzed by reverse transcription quantitative polymerase chain reaction (RT-qPCR). The primers information are listed in Table 1.The mRNA expression of MUC2 was significantly downregulated in the DSS group compared with the Control group (Figure 5A). Compared with the DSS group, the 5-ASA, *L. plantarum* AR113, *L. plantarum* Δ*bsh* 2, and *L. plantarum* Δ*bsh* 4 groups showed upregulated mRNA expression of MUC2. However, the mRNA expression of MUC2 in the *L. plantarum* Δ*bsh* 1, *L. plantarum* Δ*bsh* 13, *L. plantarum* Δ*bsh* 132, and *L. plantarum* Δ*bsh* 134 groups was not significantly different from that in the DSS group.

The relative expression levels of TJ-related genes, namely, those encoding ZO-1, occludin, and claudin, in the mouse colon tissues were assessed to investigate the integrity of the intestinal epithelial barrier and epithelial structure. The DSS group showed downregulated mRNA expression of ZO-1, occludin, and claudin relative to the Control group. In contrast, 5-ASA and *L. plantarum* AR113 treatments alleviated the DSS-induced downregulation of occludin, ZO-1, and claudin expression (Figure 5B–5D). Similarly, the *L. plantarum* Δ*bsh* 4 treatment upregulated ZO-1 and claudin expression and the *L. plantarum* Δ*bsh* 2 treatment upregulated occludin and claudin expression relative to the DSS group. However, the TJ protein expressions in the *L. plantarum* Δ*bsh* 1, *L. plantarum* Δ*bsh* 13, *L. plantarum* Δ*bsh* 132, and *L. plantarum* Δ*bsh* 134 groups were not significantly different from those in the DSS group.

### Effect of BSH on the total bile acid and the BA-specific receptors expression

**Figure 6:**
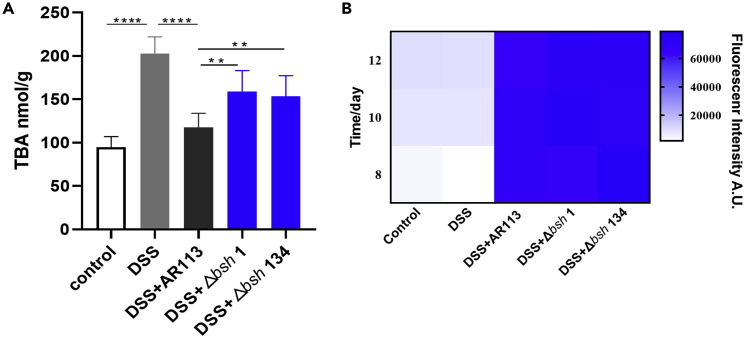
Effect of BSH on the total bile acid (A) Determination of TBA and in mice feces. (B) Determination of fluorescence intensity in mice feces. The statistical significance between the data was assessed using One-way ANOVA by Dunnett’s tests, ^∗^: p< 0.05, ^∗∗^: p< 0.01, ^∗∗∗:^p< 0.001, ^∗∗∗∗:^p< 0.0001^.^Results are express as the mean ± standard deviation for each experimental group (n = 8).

BA metabolism of IBD patients is damaged because of the impaired microbial enzyme activity, which result in the abnormal change of total bile acid (TBA). The ameliorative effect of BSH on IBD was further demonstrated by measuring the TBA in feces. The DSS group showed significantly higher of TBA compared with the Control group, the *L. plantarum* AR113 reduce effectively TBA to normal level (Figure 6A). However, the remission effect of *L. plantarum* Δ*bsh*1 and *L. plantarum* Δ*bsh*134 was significantly worse than that of the *L. plantarum* AR113 (Figure 6A).

To determine if the change of TBA is because of the strains number, red fluorescent protein (RFP) gene was successfully inserted into the *L. plantarum* AR113 and knockout strains genomes using CRISPR technology. There is no change of fluorescence intensity of *L. plantarum* AR113, *L. plantarum* Δ*bsh*1 and *L. plantarum* Δ*bsh*134 in mice feces on days 8, 10 and 12, which shows that there was no significant difference in the number of strains (Figure 6B). These findings show that the mutant strains survive in the mice to similar levels as the wild strain.

**Figure 7:**
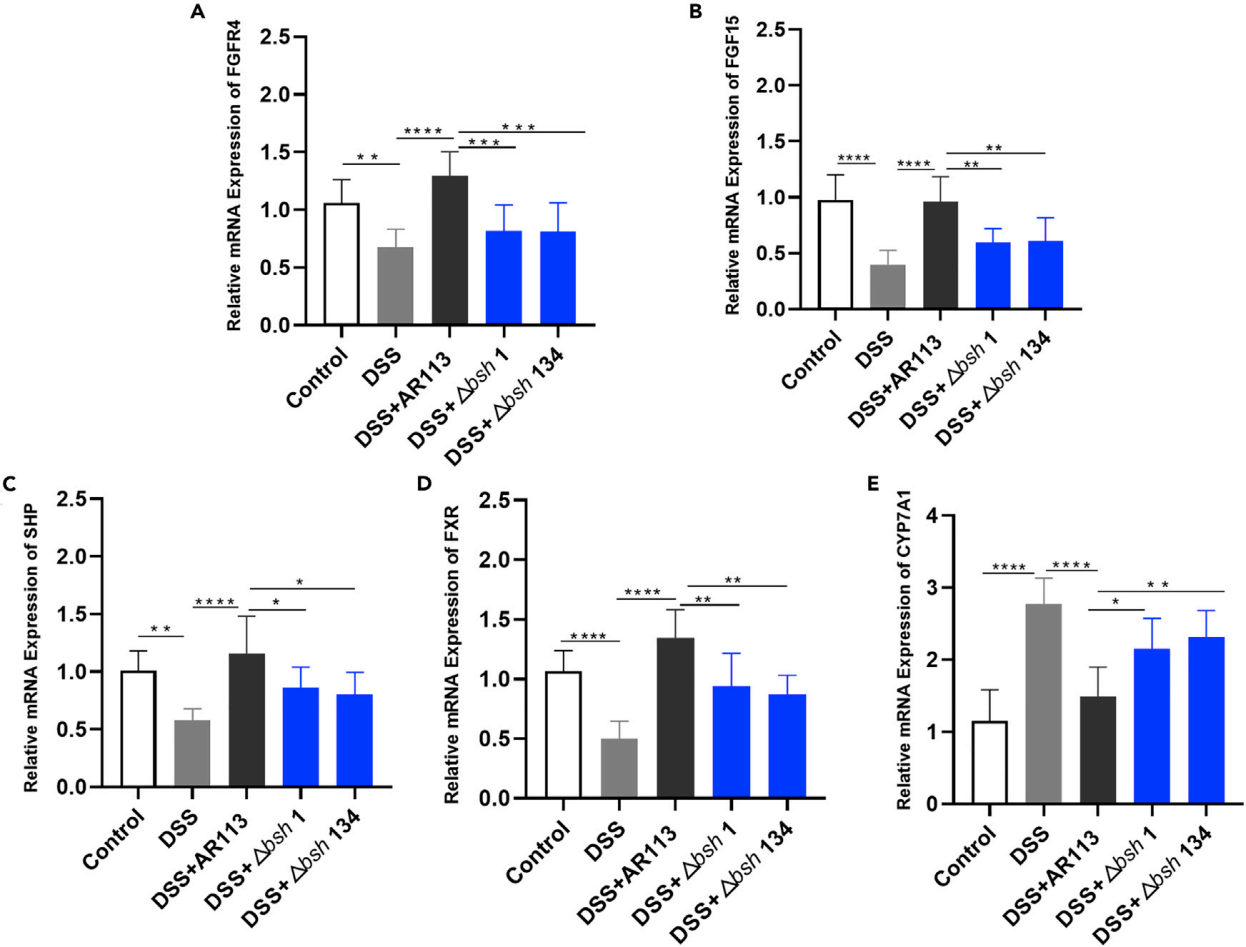
mRNA expression of BA-related genes (A) *fgfr4*, (B) *fgf15*, (C) *shp*, (D) *fxr*, (E) *cyp7a1* were quantified by RT-qPCR. The statistical significance between the data was assessed using One-way ANOVA by Dunnett’s tests, ^∗^: p< 0.05, ^∗∗^: p< 0.01, ^∗∗∗:^p< 0.001, ^∗∗∗∗:^p< 0.0001^.^ The data are expressed as the mean ± standard deviation for each experimental group (n = 8 mice per group).

We further investigated the effect of BSH on the BA-specific receptors expression. Compared with the Control group, the expression of *fgfr4*, *fgf15*, *shp* and *fxr* are downregulated in DSS group significantly (Figures 7A–7D), but the expression of *cyp7a1* is significantly increased (Figure 7E). *L. plantarum* AR113 treatments, in contrast, effectively alleviated the DSS-induced downregulated of *fgfr4*, *fgf15, shp* and upregulated of *cyp7a1*, even approaching to the normal levels (Figure 7). *L. plantarum* Δ*bsh* 1 and *L. plantarum* Δ*bsh* 134 groups have a similar regulatory effect, but their effect was significantly worse than that of *L. plantarum* AR113 and was closer to that in the DSS group (Figure 7). These results show that *L. plantarum* can regulate BA-specific receptors expression BA receptor expression through BSH.

## Discussion

In our study, *L. plantarum* AR113 relieved the symptoms of DSS-induced colitis in mice by reducing DAI values, inhibiting the hyperactivated MPO activity, and increasing the colon length, but the *bsh* 1 or *bsh* 3 knockout strains did not. To the best of our knowledge, this is the first study to demonstrate probiotics can exert ameliorative effects on IBD through BSHs. BSH is a crucial enzyme that catalyzes an essential gateway reaction in BA metabolism. Our research also found that *L. plantarum* can affect total bile acid by BSH. This suggests that probiotics may influence the occurrence of inflammation by affecting BA metabolism. Many studies have suggested that BSH activity and subsequent BA modification could significantly impact the pathophysiology of metabolic diseases, such as obesity, diabetes, and atherosclerosis, through perturbations of the BA pool.^28^^,^^29^ Therefore, it is worth further studying the effect of probiotics on these metabolic diseases by changing BA and establish the corresponding relationship between BSH activity and prebiotic function.

In addition, we demonstrated the role of BSH in alleviating inflammation, providing a basis for rational and accurate screening of probiotics. To our best knowledge, few studies have examined the active component of probiotic that alleviate inflammation by constructing knockout strains. SCFAs are metabolized by gut bacteria have been shown to ameliorate inflammatory.^10^ Although the exact source for the action of SCFA are still not clear, some probiotics that can reduce inflammation do not produce SCFAs. The exact function of SCFAs produced by probiotics has not been studied by knocking out SCFAs synthetic genes. We found that BSH is the active component in alleviating inflammation through single, double, and triple *bsh* knockout strains. So BSH activity can be included in the criteria for the anti-inflammatory probiotic strain selection.

In our study, *L. plantarum* AR113 treatment significantly downregulated the expression of TNF-α, IL-6, and IL-1β and upregulated that of IL-10, however, *L. plantarum* Δ*bsh* 1 and *L. plantarum* Δ*bsh* 3 treatments did not affect the inflammatory cytokine expression. This indicated that BSH are involved in the regulation of these factors. Some studies have demonstrated that BAs inhibit the secretion of TNF-α, IL-1β, and IL-6 in macrophages, and that this downregulation is mediated by the BA-specific membrane receptor TGR5.^30^ Some studies also found that BA-dependent farsenoid X receptor (FXR) activation appears to limit mucosal inflammatory responses via reducing pro-inflammatory cytokine (e.g., IL-1β, IL-6).^31^ In this study, *L. plantarum* AR113 significantly upregulated of FXR expression, whereas *L. plantarum* Δ*bsh*1 and *L. plantarum* Δ*bsh*134 did not. This suggests *L. plantarum* AR113 may modulate inflammatory balance by regulating bile salts. Parasar et al.^17^ identified altered BSH activities in a murine model of IBD, which led to changes in BA metabolism that could impact host metabolism and immunity. They also reported that Firmicutes-derived BSH was significantly reduced in IBD patients relative to healthy individuals. This suggests from another aspect that oral administration of *L. plantarum* can regulate BA-dependent immune responses to alleviate DSS-induced colitis through the BSH activity.

### Limitations of the study

This study focuses on the role of BSH in studying the ameliorative effects of BSH in enterohepatic circulation and the ameliorative effects of BSH on the disease severity of mice with DSS-induced colitis, whereas the mechanism of BSH alleviating the colitis needs more detailed research. It is well known that a balanced and intact intestinal environment is closely related to intestinal health and disease treatment; it would thus be interesting to investigate the changes of intestinal flora community, abundance changes and potential mechanisms.

## STAR★Methods

### Key resources table

**Table:** 

REAGENT or RESOURCE	SOURCE	IDENTIIER
**Bacterial and virus strains**		

*Lactiplantibacillus plantarum*	Shanghai Engineering Research Center of Food Microbiology, University of Shanghai for Science and Technology	AR113

**Chemicals, peptides, and recombinant proteins**		

Hieff qPCR SYBR Green Master Mix	Yeasen Biotechnology, China	11201ES03
HiScript Ⅲ RT SuperMix for qPCR	Vazyme Biotech, China	R323-01
Total RNA Extractor (TRIzol)	Shenggong bio, China	B511311-0100
5-Aminosalicylic acid	Shyuanye	S30083

**Critical commercial assays**		

TBA determination kit	(Tongwei, China)	TWP110234
MPO Detection Kit	Nanjing Jiancheng Bioengineering Institute, China	A044-1-1

**Oligouncleotides**		

See Table 1 for primers	N/A	N/A

### Resource availability

#### Lead contact

Further information and requests for resources and reagents should be directed to and will be fulfilled by the corresponding author, Professor Guangqiang Wang (1015wanggq@163.com).

#### Materials availability

This work did not generate new unique reagents. Primers sequences used were provided in Table 1, and available upon request to the corresponding author.

### Experimental model and subject details

#### Strains and culture conditions

*L. plantarum* AR113 and its seven *bsh*mutant strains were obtained from Shanghai Engineering Research Center of Food Microbiology, University of Shanghai for Science and Technology (Shanghai, China).^32^ All strains were anaerobically cultured in deMan, Rogosa, and Sharpe (MRS) medium at 37°C for 16h before centrifugation (3,000 × *g* for 3minat 4°C). The cell pellets were washed twice with phosphate buffer solution (pH 7.4) and then resuspended at a density of approximately 5 × 10^9^CFU/mL, which was determined by colony counting on MRS plates, for the following experiments. Each mouse was orally administered with 1 x10^9^ CFU of the strains in 0.2mL (5 × 10^9^CFU/mL) by oral gavage daily from day 5 to day 12 of the experiment.

#### Animals and experimental design

Six-week-old male SPF C57BL/6 mice (weight, 18–20 g) were purchased from Shanghai SLRC Laboratory Animal Co. Ltd. (Shanghai, China). The mice were housed at a constant temperature of 23 ± 2°C with a 12-h dark/light cycle and given *ad libitum* access to standard chow and water at all times. All mice were allowed at least 1 week to adapt to the experimental environment before conducting the following experiments. The experimental procedures were performed in accordance with the institutional and governmental regulations on the ethical use of experimental animals.

The mice were randomly divided into 11 groups of eight (Figure 1). The Control group was given free access to sterile water and fed a normal diet for the whole experimental period (12 days). The DSS group was given free access to 2.5% DSS-containing drinking water for the first 7 days and then fed normal diet and normal water for the next 5 days of the experiment. The DSS-treated mouse groups of *L. plantarum* AR113 and its seven *bsh*mutant derivatives were administered the corresponding strains (0.2 mL containing 5 × 10^9^CFU/mL) once a day by gavage from day 5 to day 12. The 5-ASA group of DSS-treated mice was administered 5-ASA (0.2mL) once a day by gavage from day 5 to day 12.

### Method details

#### Evaluation of the DAI

The DAI scores were calculated using a scoring system that takes into account weight loss, stool consistency, and hematochezia.^20^ First, an occult stool blood test was performed using the Occult Blood Kit (Beisuo, Zhuhai, China). Then, the following scoring criteria were used to record the DAI scores: A: weight loss (0, no loss; 1, 1%–5% loss; 2, 6%–10% loss; 3, 11%–15% loss; and 4, >15% loss); B: stool consistency (0, normal; 2, loose stools; 3 and 4, diarrhea); C: Occult blood or gross bleeding (0 and 1, negative; 2 and 3, hemoccult positive; and 4, gross bleeding).

#### Assessment of MPO activity

The colonic MPO activity in each group of mice was determined using an MPO test kit (Nanjing Jiancheng Co., Ltd., Nanjing, China) according to the manufacturer’s instructions. The colon tissue samples were prepared as follows: colon tissue was accurately weighed, and then physiological saline was added as the homogenization medium in a weight-to-volume ratio of 1:19. A 5% tissue homogenate was then prepared in a tissue-dispersing machine for MPO activity determination. MPO activity was measured in U/g of fresh colon tissue, with one unit of MPO defined as the amount required to degrade 1.0 μmol of hydrogen peroxide per minute at 37 °C.

#### H&E staining

The colon morphology and histopathological lesions were assessed with H&E staining (Murthy, Cooper, & Shim, 1993). The colon tissues were first cut into 5-mm-thick slices. The slices were then fixed in neutral buffered formalin for 24 h, dehydrated with graded alcohol (75%–100%) solutions, and then embedded in paraffin wax for H&E staining. The severity of colonic histological injury in each mouse was scored using a modified scoring system that took into account the degree of inflammation, mucosal damage, crypt damage, and range of pathological changes.

#### RNA extraction and RT-qPCR

Total RNA was extracted from each colon tissue sample using TRIzol reagent (Shenggong bio, Shanghai, China). cDNA was then prepared by reverse transcription using a HiScript Ⅲ RT SuperMix for qPCR (Vazyme Biotech, China) and amplified by RT-qPCR using Hieff qPCR SYBR Green Master Mix (Yeasen Biotech, China) and the appropriate primers (Table 1). The conditions were 40 cycles of 95 °C for 30 s, 95 °C for 5 s, and 60 °C for 30 s. The primers used in this study were synthesized by Beijing Genomics (Shanghai, China). The RNA expression levels of the relevant genes in each group were measured using the 2^-ΔΔCt^ method, where Δ Ct represents the difference in the Ct values between the target gene and the β-actin reference gene. The β-actin mRNA levels in the test groups are expressed as the ratio of its expression in the test group relative to that in the DSS group.

#### Fecal TBAs concentration analysis

Faecal samples were prepared for TBA analysis. Mice faeces that were stored at -80 °C were thawed in an ice bath to reduce sample degradation, 100 mg/mL of feces in each group were homogenate with ethanol, which were centrifuged at 4 °C for 10minat 12000 rpm, and the supernatant were collected and added to the reagent according to the instructions of TBA determination kit (TONGWEI, CHINA), the optical density at 405 nm was measured after 3 min for reaction, and content of TBA was calculated by the following equation.

$$TBA=C_{1}x\frac{A_{2}-A_{0}}{A_{1}-A_{0}}x\frac{V}{W}$$

in which, C1 is the standard quality concentration, A2 represents the absorbance at time t = 3 min and A1 represents the absorbance at t = 1.5 min, and A0 represents the optical density of blank. V is the volume of the ethanol, W is the weight of sample.

#### Faecal fluorescence intensity analysis

As described by Qin,^33^ red fluorescent protein (RFP) gene was inserted into *L. plantarum* AR113, *L. plantarum* Δ*bsh* 1, *L. plantarum* Δ*bsh* 134 with the CRISPR/Cas 9 gene editing tool. The upstream, downstream homologous arms of the insert plasmids and red fluorescent protein gene (RFP) were amplified by PCR using the *L. plantarum* AR113 genome and pIB184-RFP, which were ligated to the sgRNA amplified by PCR using plasmid pHSP01 as template to obtain up-RFP-down-sgRNA fragment. The fragment was cloned with the editing plasmid pHSP01 skeleton (digested by ApaI and XbaI) by one-step cloning to obtain the insertion plasmid, and the insertion plasmid was then introduced into *L. plantarum* AR113, *L. plantarum* Δ*bsh* 1 and *L. plantarum* Δ*bsh* 134, cultured at 37 °C for 48 h and screened in erythromycin. The used primer RFPinsert was listed in Table 1.

The mice were randomly divided into 5 groups (n=8). The control group was given free access to sterile water and fed a normal diet for the whole experimental period (12 days). The DSS group was given free access to 2.5% DSS-containing drinking water for the first 7 days and then fed normal diet and normal water for the next 5 days of the experiment. The DSS-treated mouse groups of *L. plantarum* AR113-RFP, *L. plantarum* Δ*bsh* 1-RFP and *L. plantarum* Δ*bsh* 134-RFP with 1х10^9^ CFU, once a day by gavage from day 5 to day 12. Faecal samples were collected on day 8, 10 and 12, and weighed as soon as possible to dissolved into 0.6% faecal homogenate in sterile water. The supernatant was collected after centrifugation at 4000g for 10 min, subsequently, 200 μL of the supernatant was added to a 96-well plate, in addition to sterile water as the standard, the absorbance (excitation wavelength, 600 nm; emission wavelength, 635 nm) was measured using a fluorescence spectrophotometer (SpectraMax i3x, Molecular Devices, San Jose, CA, USA).

### Quantification and statistical analysis

Statistical analyses were performed using SPSS software (SPSS Inc, Chicago, U.S.A.) or GraphPad Prism 5 (GraphPad Software, Inc, San Diego, U.S.A.). All data are presented as the mean ± standard deviation (n = 8 per group). The statistical significance of the results was analyzed by a one-way analysis of variance followed by a Tukey’s post hoc test. A p-value < 0.05 was considered significant.

## Data Availability

The data reported in this paper will be shared upon request to the lead corresponding author (1015wanggq@163.com). This paper dose not report original code. Any additional information required to reanalyze the data reported in this paper is available from the lead contact upon request.
