# Supplementary material for: Bile salt hydrolase of Lactiplantibacillus plantarum plays important roles in amelioration of DSS-induced colitis
Source: iScience. 2023 Feb 13;26(3):106196. doi: 10.1016/j.isci.2023.106196 (PMC9988676; doi:10.1016/j.isci.2023.106196)
Supplement: Document S1. Figures S1 [file mmc1.pdf]

iScience, Volume *26*

Supplemental information

Bile salt hydrolase of *Lactiplantibacillus plantarum* plays important roles in amelioration of DSS-induced colitis

Xin Feng, Zichen Bu, Hongyu Tang, Yongjun Xia, Xin Song, Lianzhong Ai, and Guangqiang Wang

Supplementary Figure

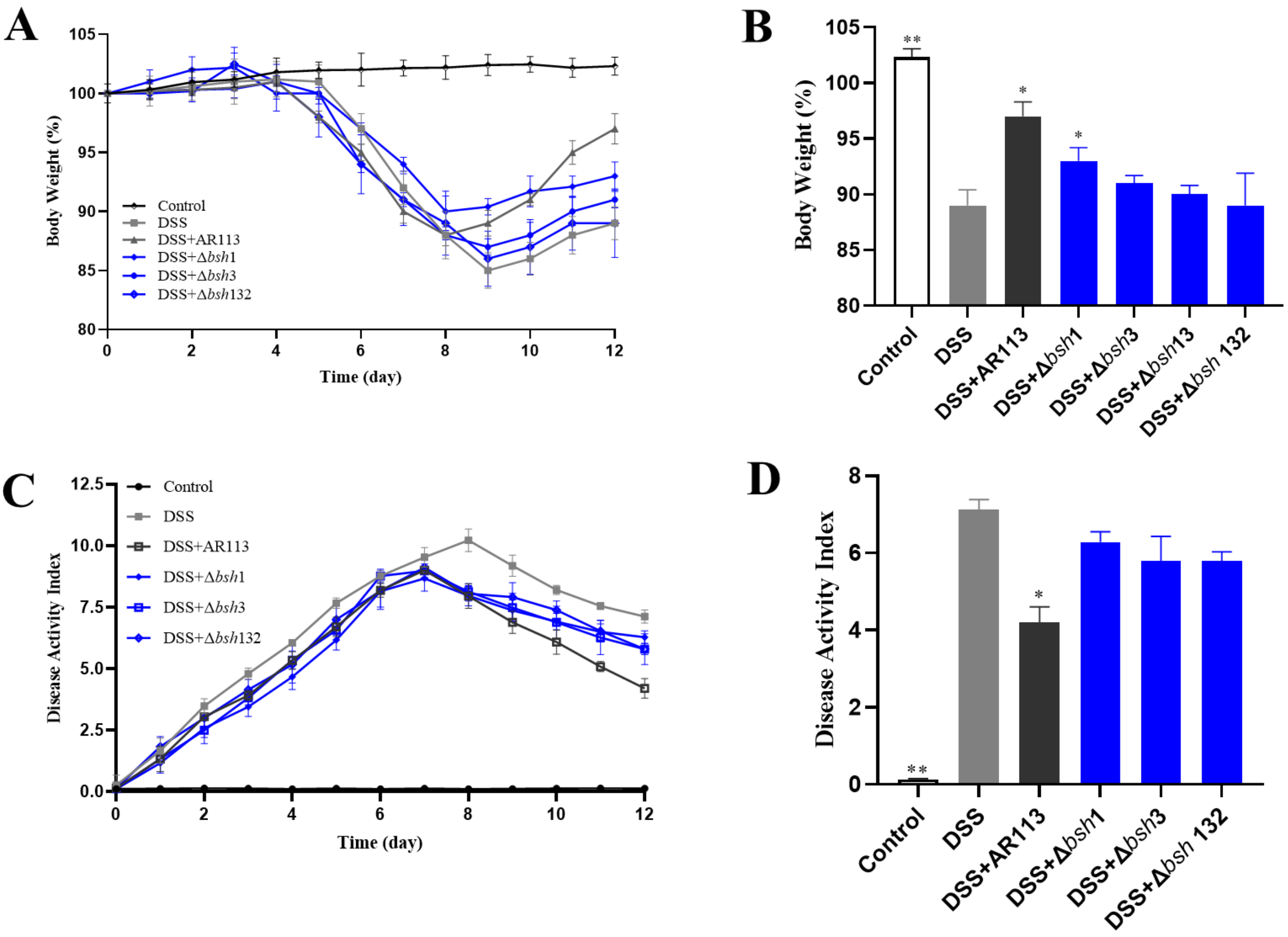

Figure S1 Physical and chemical indicators of colitis in mice, related to Figure 2. (A) Body weight, (B) body weight at day 12, (C) disease activity index (DAI), (D) DAI at day 12. The blue bar indicates the group with bsh 1 or bsh 3 knocked out. The statistical significance between the data was assessed using One-way ANOVA by Dunnett's tests, *: $p < 0.05$, **: $p < 0.01$. All data are presented as the mean ± standard error of the mean (n = 8 mice per group).
